# Sleep Quality and Subjective Cognitive Decline among Older Adults: The Mediating Role of Anxiety/Depression and Worries

**DOI:** 10.1155/2024/4946303

**Published:** 2024-05-07

**Authors:** McKenna Sun, Qianqian Zhang, Yifei Han, Jianghong Liu

**Affiliations:** ^1^University of Pennsylvania, Philadelphia, PA 19104, USA; ^2^Northeastern University, Boston, MA 02115, USA

## Abstract

**Background:**

Subjective cognitive decline (SCD) in older individuals has been implicated as a possible precursor to Alzheimer's disease. Poor sleep quality and anxiety/depressive symptoms have been linked to the progression of SCD, but these associations and older adults' worries have yet to be fully established in the Chinese older adult population, which is one of the largest in the world. The aim of this study was to explore the relationship between sleep quality, anxiety/depression symptoms, and worries, and SCD prevalence among Chinese community-dwelling older individuals.

**Methods:**

A total of 707 adults aged between 60 and 99 from Shanghai, China, completed self-report questionnaires that covered their cognitive and mental well-being, as well as demographic information. Subjective cognitive decline (SCD) was evaluated using the memory/cognition syndromes of the old adult self-report (OASR). Sleep quality, anxiety/depression, and worries were measured from their respective sections of the OASR.

**Results:**

The general linear regression models showed that poorer sleep quality was associated with an increased prevalence of anxiety/depression symptoms, worries, and SCD among older adults. As suggested by the mediation analysis, anxiety/depression and worries were significant mediators in the relationship between sleep quality and SCD prevalence, and these two factors also have a serial mediation effect between sleep quality and SCD prevalence.

**Conclusions:**

Poorer sleep quality is associated with a higher rate of SCD among older adults, and a higher prevalence of anxiety/depression and worries mediate this relationship, suggesting possible mechanism pathways that lead to SCD. These factors may provide the basis for early, targeted interventions for older adults' mental health preservation and improved quality of life.

## 1. Introduction

Intact cognitive function is crucial to the standard of living for individuals progressing from middle to late adulthood. Subjective cognitive decline (SCD) is the self-reported decline of memory and increased confusion in individuals, and it has emerged as a growing issue as demographics skew towards older populations around the world [1, 2]. In 2016, over 8.5% of the world population was over the age of 65, and this percentage is expected to grow to 17% by 2050, representing an increase from 617 million to 1.7 billion individuals [3]. Furthermore, China is expected to gain almost 200 million older individuals alone in the next 20 years [4]. SCD has gained traction in recent years as a possible precursor to Alzheimer's disease [5]. Longitudinal studies report a higher incidence of and faster conversion rates to objective, later stages of cognitive decline, such as mild cognitive impairment and Alzheimer's disease, in adults with SCD compared to their cognitively intact counterparts [6, 7]. This warrants SCD as an important stage to explore in older adults for possible early Alzheimer's disease interventions.

In recent years, poor sleep quality has been implicated as a possible risk factor in the progression of SCD [8]. Cross-sectional studies in Korean populations have shown that poorer sleep quality is associated with an increased prevalence of and more severe SCD symptoms for both genders and among various age groups [9–11]. A longitudinal study by Zhao et al. found support for the link between insomnia and SCD in Canadian middle-aged and older adults [12]. Beyond its association with SCD, poor sleep quality is possibly associated with a higher incidence of objective cognitive function issues although the findings are mixed. Siddarth et al. found objective memory differences between adults who reported SCD and adults without SCD [13]. However, Exalto et al. reported that poorer sleep quality in adults was associated with more severe SCD symptoms but not cognitive testing, MRI, or cerebrospinal fluid biomarker differences [14]. In addition, there is a lack of SCD and sleep quality literature for Chinese populations, which is necessary considering the increasing incidence of SCD in China. While Wang et al. found a correlation between poor sleep and SCD in Western Chinese adults, it may not be fully generalizable to the Chinese population [15]. Therefore, additional research is necessary to corroborate these findings and apply to more older Chinese adults.

Increased anxiety and depressive symptoms have also been associated with the prevalence of SCD. Depressive symptoms and SCD each independently contribute as risk factors to more severe neurocognitive disorders, and their comorbidity greatly increases this risk [16]. Adults with SCD experienced greater perceived stress and had increased anxiety/depressive symptoms during COVID-19-related confinement [17]. A cross-sectional study across 47 middle-to low-income countries indicated that depressive symptomatology was associated with more severe SCD across age, gender, and education groups [18]. In addition, patients who reported SCD tend to be older, have less education, and have higher levels of anxiety/depression although these associations are not necessarily correlated with objective cognitive decline [19]. Higher levels of worry are also associated with greater amyloid-*β* concentration in the frontal lobe, a likely precursor to Alzheimer's disease, in older German adults [20]. However, there remains limited research on how the trait of worry is associated with SCD; while this trait is often conflated with anxiety/depression, it differs in that anxiety/depression often revolves around negative self-directed thinking, while worries predominantly originate from external factors, such as environmental and interpersonal stressors. Targeting external stress factors is crucial to developing and delivering cognitive therapies that may improve older individuals' quality of life.

With regards to the connection between sleep quality and anxiety/depression in older adults, there is a well-established negative correlation between the two variables. Depressed older individuals experience objectively poorer sleep and exhibit increased nocturnal awakening time [21]. The two factors play into one another as well—older individuals who are anxious and/or depressed are more likely to complain of insomnia, and in turn, insomnia increases the risk for psychiatric disorders [22]. In a cross-sectional study of southern Chinese nursing home residents, almost 70% of the residents experienced poor-quality sleep. These residents showed increased depressive symptomatology, indicating poor-sleep quality as a marker for depression [23]. For northern Chinese nursing home residents, factors such as loneliness and self-care abilities significantly affected depression in those with poor sleep quality [24]. In addition, the combination of poor sleep quality and anxiety/depression has been implicated in cognitive decline. Several studies have shown a possible mediating role of sleep quality between anxiety/depression and cognitive decline, though the findings are limited. Liu et al. indicated only a partial mediating effect from poor sleep quality while Wu et al. showed that sleep quality mediation existed only for males [25, 26]. However, it is vital to examine anxiety/depression and worries as potential mediators between sleep and SCD because poor sleep has been implicated to increase negative mood states [27].

In terms of the mediating effects of anxiety/depression and worries, recent research suggests that anxiety/depression mediates the relationship between sleep quality and SCD. Xiao et al. and Xu et al. both found that poor sleep quality and SCD or cognitive complaints were mediated by depression and anxiety symptomology in older Chinese adults [28, 29]. However, a relationship between sleep quality and SCD with worries as a possible mediator has yet to be established. The primary objective of the present study is to fill the existing gaps in the literature by investigating the relationship between sleep quality, anxiety/depression symptoms, worry traits, and SCD in a substantial sample of older Chinese adults. We hypothesized that poor sleep quality is associated with subjective cognitive decline among older adults and is mediated by anxiety/depression symptoms and worries.

## 2. Materials and Methods

### 2.1. Study Population

A total of 755 elderly individuals, aged 60 and above, without significant mental or physical impairments, were included in this community sample. The recruitment of this participant group was undertaken by the nursing research teams from Shanghai Longhua Hospital and Shanghai Shuguang Hospital, with a focus on 25 local communities in Shanghai, China. This was done through a combination of convenience and community sampling methods in November 2017 [30]. Out of the initial group, 707 participants successfully completed the survey and they received compensation of 100 Chinese Yuan each.

The survey was administered by a team of 150 interviewers, composed of 40 nursing supervisors from Shanghai Longhua Hospital and 110 interns from Shanghai Shuguang Hospital. Prior to conducting the interviews, comprehensive training workshops were conducted to ensure the standardization of interview procedures. During the interviews, the participants were presented with a written survey to follow, while the interviewers verbally presented each item and recorded the participants' responses on a separate copy. If participants were capable of independently completing the items, they were given the option to write their own answers. Detailed sampling and recruitment methods were provided in our previous publication [30].

All participants provided written informed consent, and the research was conducted with approval from the Institutional Review Boards of both Shanghai Shuguang Hospital and Shanghai Longhua Hospital (Ethics approval number: 2020-GZR-02-053X).

### 2.2. Measure

The assessment of anxiety/depression symptoms, worry levels, and subjective cognitive decline (SCD) in these older adults was conducted using the memory/cognition syndrome scales of the older adult self-report (OASR) and the older adult behavior checklist (OABCL). The OASR is a standardized instrument developed by Achenbach et al. for older adults to self-report their adaptive functioning; behavioral, emotional, and social problems, as well as their substance use (i.e., cigarettes, alcohol, and other drugs) [31]. Likewise, the OABCL was developed by Achenbach et al. for people close to the adults to report the adults' functioning, allowing for comparisons between their self-perception and others' perceptions [31]. Previous research has demonstrated the cross-cultural applicability of the OASR across 20 different societies, including China, Brazil, and Germany [32]. Specifically, the Chinese version of the OASR and OABCL has been validated for use among Chinese-speaking older adult populations in clinical and research settings, enabling the assessment of mental health in this demographic [30, 32].

Anxiety/depression symptoms were measured using the anxious/depressed syndrome scale of the OASR, which encompasses 20 items, such as “feels worthless” and “self-conscious”. Worry levels were evaluated using the worries scale of the OASR, which covers eight items, such as “feels burdensome” and “worries about family”. Participants provided ratings for all items on a three-point scale (0 = “not true,” 1 = “sometimes true,” and 2 = “often true”), which reflected the participants' experiences over the preceding 2 months.

Sleep quality was measured using the Pittsburgh Sleep Quality Index (PSQI), which consists of 19 items that cover 7 subscales: sleep quality, sleep latency, sleep duration, sleep efficiency, sleep disturbances, use of sleep medications, and daytime dysfunction. The PSQI has been demonstrated to be reliable and valid in various Chinese populations, including older populations [33–35].

Subjective cognitive decline (SCD) was evaluated in accordance with the scoring methodology mentioned above, utilizing the memory/cognition problems syndrome scale within the old adult self-report (OASR). This scale comprises nine items that vary from “cannot concentrate” to “forgets names.” Scores for SCD were derived by summing the responses for each item within the syndrome, which were presented in a normalized T-score format (*M* = 50, SD = 10), and higher scores indicated a greater degree of subjective cognitive decline [36].

Demographic variables encompassed age, gender (male or female), education level (high education: high school equivalent and beyond and low education: incomplete high school education), income level (high income: total monthly income exceeded RMB 3000 and low income: total monthly income was below RMB 3000), and marital/living status (lives with spouse/partner: married and cohabiting with spouses or unmarried but cohabiting with partners, and other: unmarried, divorced, widowed, or not cohabiting with a spouse/partner over the last 2 months). In terms of age stratification for analysis, the sample was divided into two distinct categories: the young-old group, encompassing individuals aged 60–74 and the old-old group, encompassing those aged 75 and above. These factors were all controlled for in the subsequent mediation models.

### 2.3. Data Analyses

The current study includes 707 data, excluding 8 individuals who had no OASR record, leaving 699 valid participants. Sample characteristics were summarized by descriptive statistics, including the sample count, percentage, mean, and standard deviation, as appropriate. With adjustments for age, gender, education, income, and marital status, generalized linear models (GLMs) were applied to evaluate the independent effects of the following relationships: (1) sleep quality on anxiety/depression, (2) sleep quality on worries, (3) sleep quality on SCD, and (4) sleep quality, anxiety/depression, and worries on SCD. Separated GLMs were created for both self-reported and other-reported SCD. The mediation analyses were performed in three stages to identify whether anxiety/depression and worries mediated the relationship between sleep quality and SCD among old adults. A single mediation model investigated the mediation effect separately for anxiety/depression and worries. Nonparametric bootstrapping analyses were deployed to test the mediate effects of anxiety/depression and worries as parallel mediators of the relationship between sleep quality and SCD. Serial mediation models were performed to test the serial causal chain of the two mediators using PROCESS 4.3 (Model 6) [37]. Sleep quality, anxiety/depression, worries, and SCD were standardized (*t* score) before running the analyses. Statistical analyses were processed by RStudio. *p* values less than 0.05 were considered statistically significant.

## 3. Results

### 3.1. Sample Characteristics

The basic demographic characteristics of old adults stratified by age and gender were summarized in Table 1. Overall, about three fourths (*N* = 510) of old adults were young-old adults (aged between 60 and 74) and one fourth were old-old adults (aged above 74). The gender consisted of 45% (*N* = 307) males and 55% (*N* = 375) females. The mean age of all participants was 69.68 (SD = 7.46), 66.14 (SD = 4.37) for the young-old group and 80.17 (SD = 4.37) for the old-old group. A total of 78 (12%) old adults reported having bachelor's and higher degrees, 179 (26%) old adults obtained high-school degrees, and the rest were at middle school and lower education levels (*N* = 425, 62%). High-income participants (*N* = 336) accounted for 49% of the whole sample, and there was a similar ratio of low-income participants (*N* = 346). About three fourths (*N* = 492, 72.14%) of old adults lived with their spouse/partner in the past 2 months, and 190 (27.86%) old adults were in “other” marital status.

### 3.2. Associations between Sleep Quality, Anxiety/Depression, and Worries

As demonstrated in Table 2, controlling for age, gender, education, income, and marital status; sleep quality was positively associated with anxiety/depression and worries, suggesting that old adults who had poorer sleep quality tended to have higher prevalence of anxiety/depression (*β* = 0.49, *p* < 0.001) and worries (*β* = 0.36, *p* < 0.001).

### 3.3. Association between Sleep Quality and SCD

Significant associations between sleep quality and SCD in both forms were observed in GLMs (Table 3). After controlling for confounding variables (age, gender, education, income, and marital status), sleep quality was positively associated with higher scores in SCD, showing that the old adults who had poor sleep quality (*β*_self‐reported_=0.24, p < 0.001;  *β*_other‐reported_=0.24, p < 0.001) tended to have a higher prevalence of SCD problems.

### 3.4. Associations between Sleep Quality, Anxiety/Depression, Worries, and SCD

Anxiety/depression and worries were associated with SCD (Table 4). After controlling for possible confounding variables, old adults who have more anxiety/depression problems (*β*_self‐reported_=0.31, *p* < 0.001;  *β*_other‐reported_=0.30, *p*<0.001) and higher prevalence of worries (*β*_self‐reported_=0.21, *p* < 0.001;  *β*_other‐reported_=0.24, *p*<0.001) tended to have higher scores in both self-reported and other-reported SCD. Sleep quality showed an insignificant association with self-reported and other-reported SCD after controlling all other variables (*β*_self‐reported_=0.01, *p*=0.599;  *β*_other‐reported_=0.03, *p*=0.257).

### 3.5. Mediation Roles of Anxiety/Depression and Worries between Sleep Quality and SCD

#### 3.5.1. Single Mediation Analysis

**Figure 1:**
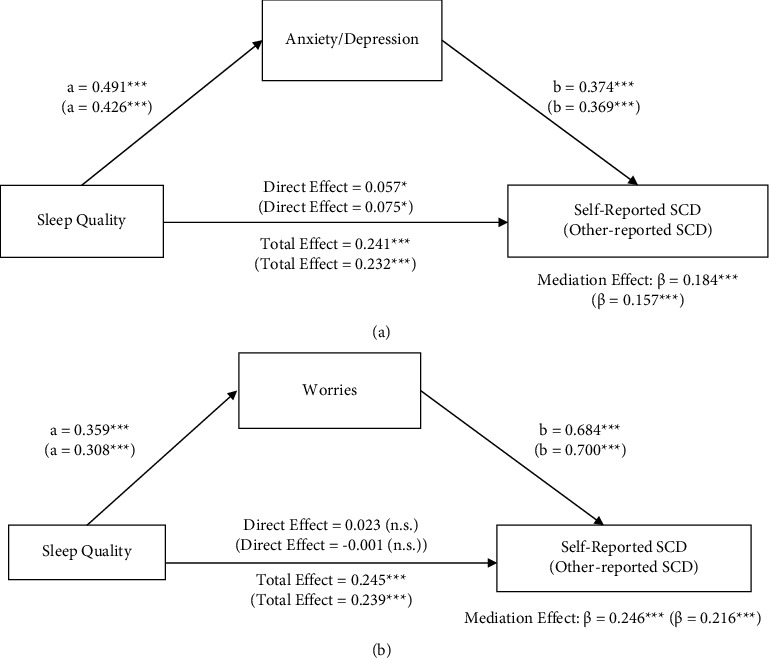
(a) Mediation role of anxiety/depression between sleep quality and self-reported SCD. *Note*. Mediation analyses were performed with 1000 bias-corrected bootstrapped samples. Unstandardized regression coefficients reported from self-reported SCD models were out of brackets and other-reported coefficients were in brackets. The models are controlled for age, gender, education, income, and marital status. ^*∗*^=*p* < 0.05, ^*∗∗∗*^=*p* < 0.001. (b) Mediation role of worries between sleep quality and self-reported SCD. *Note*. Mediation analyses were performed with 1000 bias-corrected bootstrapped samples. Unstandardized regression coefficients reported from self-reported SCD models were out of brackets and other-reported coefficients were in brackets. The models are controlled for age, gender, education, income, and marital status. ^*∗∗∗*^=*p* < 0.001, n.s. = not statistically significant.

The mediation roles of anxiety/depression and worries are shown separately in Figures 1(a) and 1(b). As shown in Figure 1(a), anxiety/depression partially mediated the association between sleep quality and SCD (mediation effect_self‐reported_=0.184, *p* < 0.001). The significant indirect effect explained 76% of the total effect of sleep quality on SCD, and the direct effect of sleep quality was significant (*β*_self‐reported_=0.057, *p* < 0.05). The mediation effect of worries (mediation effect: *β*_self‐reported_=0.246, *p* < 0.001) was significant, while the direct effect of sleep quality (*β*_self‐reported_=0.023) on SCD was not significant. Therefore, worries fully mediated the relationship between sleep quality and SCD.

#### 3.5.2. Parallel Mediation Analysis

**Figure 2:**
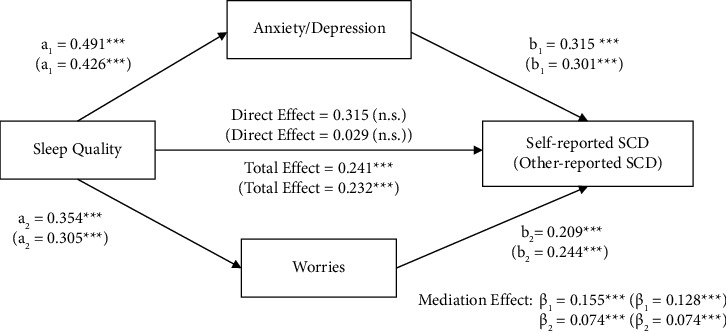
Parallel mediation model. Mediation effects of sleep quality on SCD through anxiety/depression and worries. *Note*. Mediation analyses were performed with 1000 bias-corrected bootstrapped samples. Unstandardized regression coefficients for self-reported SCD as outcome were out of brackets and coefficients for other-reported SCD as outcome were in brackets. The models are controlled for age, gender, education, income, and marital status. ^*∗∗∗*^=*p* < 0.001, n.s. = not statistically significant.

As shown in Figure 2, the mediation effects of anxiety/depression (*β* = 0.155, *p* < 0.001^*∗∗∗*^) explained 63% of the total effect and worries (*β* = 0.074, *p* < 0.001^*∗∗∗*^) explained 31% of the total effect while controlling for confounding variables. The direct effect of sleep quality on SCD was insignificant. Both anxiety/depression and worries fully mediate the relationship between sleep quality and SCD, suggesting that old adults who had poorer sleep quality were more likely to have higher levels of anxiety/depression and worries. In addition, old adults with a higher prevalence of anxiety/depression and worries tended to report higher levels of SCD symptoms.

#### 3.5.3. Serial Mediation Analysis

**Figure 3:**
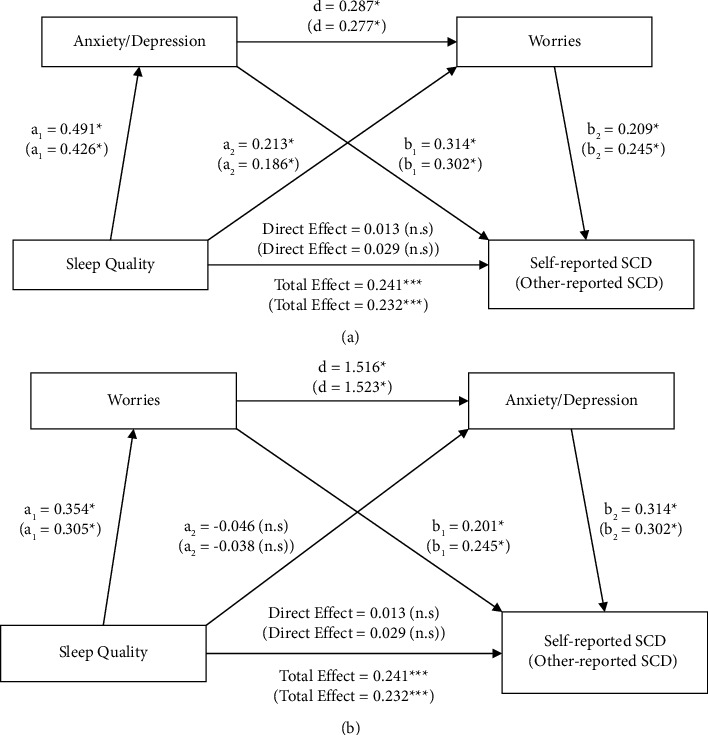
(a) Serial mediation model 1 for SCD. *Note.* Serial mediation analyses were performed with 1000 bias-corrected bootstrapped samples. Unstandardized regression coefficients of self-reported SCD as outcome were out of brackets and coefficients for other-reported SCD as outcome were in brackets. Model tests the mediation effect of three pathways: (a) sleep quality ⟶ anxiety/depression ⟶ SCD; (b) sleep quality ⟶ worries ⟶ SCD; and (c) sleep quality ⟶ anxiety/depression ⟶ worries ⟶ SCD. Numbers associated with a, b, c, and d are unstandardized regression coefficients. The models are controlled for age, gender, education, income, and marital status. ^*∗*^=*p* < 0.05, ^*∗∗∗*^=*p* < 0.001, n.s. = not statistically significant. (b) Serial mediation model 2 for SCD. *Note*. Serial mediation analyses were performed with 1000 bias-corrected bootstrapped samples. Unstandardized regression coefficients of self-reported SCD as outcome were out of brackets and coefficients for other-reported SCD as outcome were in brackets. Model tests the mediation effect of three pathways: (a) sleep quality ⟶ anxiety/depression ⟶ SCD; (b) sleep quality ⟶ worries ⟶ SCD; and (c) sleep quality ⟶ worries ⟶ anxiety/depression ⟶ SCD. Numbers associated with a b, c and d are unstandardized regression coefficients. The models are controlled for age, gender, education, income, and marital status. ^*∗*^=*p* < 0.05, ^*∗∗∗*^=*p* < 0.001, n.s = not statistically significant.

The serial mediation revealed a causal chain linking of mediators (anxiety/depression and worries) in two specified directions (Figure 3(a) and 3(b)). The bootstrapped results of each path are detailed in Table 5. The results of serial mediation models showed that anxiety/depression and worries fully mediated the relationship between sleep quality and SCD (total mediation effect = 0.229, *p* < 0.05), which explained 95% of the total effect (total effect = 0.241, *p* < 0.001). In serial model 1, the mediation effect path of sleep quality ⟶ anxiety/depression ⟶ SCD (mediation effect = 0.155) explained 64% of the total effect. The path of sleep quality ⟶ anxiety/depression ⟶ worries ⟶ SCD was significant, suggesting that poorer sleep quality leads to an increase in the prevalence of both anxiety/depression and worries, anxiety/depression can cause an increased prevalence in worries as well, and the incidence of both anxiety/depression and worries may contribute to an increase in SCD symptoms. In serial model 2, the path of sleep quality ⟶ worries ⟶ anxiety/depression ⟶ SCD yielded a high ratio of mediation effect to total effect (mediation effect = 0.169, 70% of the total effect), and the path of sleep quality ⟶ anxiety/depression ⟶ SCD was not significant. The results suggested that poor sleep quality may lead to an increase in the prevalence of worries, thereby causing a rise in anxiety/depression and, therefore, SCD symptoms.

## 4. Discussion

This cross-sectional study from Shanghai community-dwelling older adults found that self-reported anxiety/depression symptoms and worries are mediating factors between older individuals' sleep quality and SCD. Such relationships are still held even when considering other extraneous factors, such as gender, income, education, and marital status. In addition, a serial mediation effect of anxiety/depression and worries was found connecting sleep quality to SCD. These findings highlight important implications in developing more targeted interventions to slow the progression of SCD into objective cognitive deterioration.

### 4.1. Effect of Sleep on Subjective Cognitive Decline

Previous cohort studies done in other regions of China found that anxiety/depressive symptoms mediate the relationship between sleep quality and SCD, and our study findings were consistent with them, thus improving the possibility of generalizing such results to more of the Chinese population [28, 29, 38]. These findings also agree with literature on anxiety and depression independently increasing sleep issues in older adults [39–41]. In addition, the link between sleep quality and SCD is reaffirmed, despite mixed results on the subject—non-Chinese cohort studies have connected SCD with objective memory loss, but not sleep quality with objective memory loss even though sleep quality is associated with SCD [13, 14]. Nevertheless, research on such connections in Chinese populations is minimal, and our findings corroborate the connection between poor sleep and SCD in older residents of Western China [15]. Depression has also been previously suggested as a partial mediator between SCD symptoms and decreased objective memory, making our findings important as anxiety/depression may now play a key role even earlier in cognitive decline mechanisms [42]. Given the high incidence of significant depressive symptoms in Chinese older adults—up to 60.3% in some regions—targeting anxiety/depression may be particularly beneficial in reducing older adults' progression of SCD [43].

### 4.2. Worries as a Mediator

Significantly, our findings illustrate that worries are an additional mediator between sleep quality and SCD. To date, no studies have focused on worries and their impacts on regulating the mechanism between sleep and early stages of cognitive decline. Repetitive negative thinking is associated with SCD, and increased amyloid-beta loads are present in the brains of older individuals who report more worries, which possibly indicates Alzheimer's disease progression [20, 44]. It has also been suggested that poor sleep provides a pathway for beta-amyloid to accumulate in the brain [45]. This provides a possible mechanism for how worries act as a mediator between sleep quality and SCD—poor sleep incites the accumulation of beta-amyloid, which is associated with worries, and beta-amyloid is a marker for SCD and more severe forms of dementia. Thus, it is reasonable to believe that poor sleep not only acts on SCD directly as suggested by previous cross-sectional studies but also by increasing worries, which act on SCD as well [9–12]. This novel finding is beneficial in providing increased possibilities for interventional strategies beyond simply treating anxiety/depression symptoms and may improve the efficacy of current treatment strategies by targeting worries in older individuals.

### 4.3. Anxiety/Depression and Worries as Serial Mediators

In addition, our findings are the first to support a serial mediation model connecting sleep quality, anxiety/depression, worries, and SCD. To our knowledge, most studies have focused on the mediating effect of anxiety/depression between sleep quality and SCD and have not connected worries as a possible factor in the pathway. The interchangeability of anxiety/depression and worries as serial mediators, as both serial pathways were significant, expands the possible therapeutic interventions for delaying the onset of SCD. While anxiety/depression and worries are closely related, they are still inherently different in their categorization in the OASR: anxiety/depression items more often focus on negative thinking towards self while worries are directed more externally and are a reaction to environmental and relationship stressors. Due to the possible conflation between anxiety/depression and worries, there is minimal research in older populations that examines the connection between the two variables. Our differentiation between the two factors is significant because it can address older individuals' mental health in a more effective fashion: simultaneously addressing more internalizing anxiety/depression and externalizing worries may provide even better cognitive outcomes for older individuals.

### 4.4. Limitations

The interpretation of the findings should take into account the inherent limitations of the study. First, it is important to note that this cross-sectional study utilized a survey method to measure the prevalence of cognitive decline. As a result, both explanatory variables and outcomes were collected simultaneously, without a prospective or retrospective follow-up. Consequently, establishing a causal relationship based on these findings is not currently feasible. A future longitudinal study may better examine the full causality among all variables. Second, while our model considers worries to be a mediator that results in the development of SCD, other literature suggests worries are a symptom of SCD [42]. Further research may be necessary to clarify the relationship between the two factors given the general paucity of research on the possible impacts of worries on SCD. Third, it is worth noting that the OASR, the instrument employed in this study, was originally developed based on Western samples. As a result, it may not fully capture the complete range of behaviours and traits that are specific to Chinese culture and the Chinese population. Lastly, the study's community sample specifically consisted of older adults from Shanghai, a highly developed city in China. Therefore, caution should be exercised when generalizing the results to other geographic regions, especially rural areas, and regions with greater ethnic diversity. In light of these limitations, a comprehensive understanding of the study's findings necessitates careful consideration of these factors to ensure accurate interpretation and appropriate generalization.

## 5. Conclusions

By 2050, close to 20% of the global population will be above the age of 65. This looming surge in the older population spotlights a need to ensure that older individuals minimize their risk of SCD and more serious forms of dementia for a healthier aging process. Our study found an independent mediating effect and the serial mediating effect of anxiety/depression and worries on the relationship between sleep quality and SCD in an older Chinese population while controlling for extraneous factors. This is vital to addressing not only SCD prevalence but also the high rates of depression/anxiety in older Chinese adults in the status quo. More research is warranted to examine the long-term outcomes of these individuals, establish a causal relationship through a longitudinal study, and test the efficacy of possible interventions. Our current and future findings have important implications in creating targeted, personalized treatment plans for protecting the mental health of older individuals. Such interventions are vital to decreasing cognitive impairment levels and improving day-to-day functioning and interpersonal relationships for a growing population of older adults.

## Figures and Tables

**Table 1:** Sample characteristics by age and gender.

	Total (*N* = 682)	Young-old (*N* = 510)	Old-old (*N* = 172)	Males (*N* = 307)	Females (*N* = 375)
Age	69.68 (7.46)	66.14 (4.37)	80.17 (4.13)	70.69 (7.64)	68.85 (7.22)
Young-old	510 (75%)	—	—	213 (69%)	297 (79%)
Old-old	172 (25%)	—	—	94 (31%)	78 (21%)
Gender					
Male	307 (45%)	213 (42%)	94 (55%)	—	—
Female	375 (55%)	297 (58%)	78 (45%)	—	—
Education					
≥Bachelor's degree	78 (11%)	57 (11%)	21 (12%)	43 (14%)	35 (9%)
High school	179 (26%)	126 (25%)	53 (31%)	84 (27%)	95 (25%)
≤Middle School	425 (62%)	327 (64%)	98 (57%)	180 (59%)	245 (65%)
Income					
High income	336 (49%)	259 (51%)	77 (45%)	128 (42%)	208 (55%)
Low income	346 (51%)	251 (49%)	95 (55%)	179 (58%)	167 (45%)
Marital status					
Living with spouse/partner	492 (72%)	398 (78%)	94 (55%)	247 (80%)	245 (65%)
Other	190 (28%)	112 (22%)	78 (45%)	60 (20%)	130 (35%)

**Table 2:** Adjusted general linear regression for the effect of sleep quality on anxiety/depression and worries.

Variables	Anxiety/depression		Worries	
	*β* (SE)	*p* value	*β* (SE)	*p* value
Sleep quality	0.49 (0.08)	≤0.001^*∗∗∗*^	0.36 (0.03)	≤0.001^*∗∗∗*^
Age	0.05 (0.03)	0.133	0.02 (0.01)	0.131
Gender	−0.51 (0.51)	0.319	−0.63 (0.22)	0.004^*∗∗*^
Education	−0.46 (0.37)	0.218	−0.30 (0.16)	0.064
Income	−0.65 (0.52)	0.206	−0.16 (0.22)	0.477
Marital status	−2.50 (0.57)	≤0.001^*∗∗∗*^	−0.47 (0.25)	0.057

*Note. β* = the change in anxiety/depression or worries while the variable increased by one unit, controlled for all other variables. SE = standard error. ^*∗∗*^=*p* < 0.01, ^*∗∗∗*^=*p* < 0.001.

**Table 3:** Adjusted linear regression for the effect of sleep quality on SCD.

Variables	Self-reported SCD		Other-reported SCD	
	*β* (SE)	*p* value	*β* (SE)	*p* value
Sleep quality	0.24 (0.04)	≤0.001^*∗∗∗*^	0.24 (0.04)	≤0.001^*∗∗∗*^
Age	0.04 (0.02)	0.020^*∗*^	0.03 (0.02)	0.089
Gender	−0.43 (0.24)	0.075	−0.13 (0.25)	0.612
Education	−0.13 (0.18)	0.448	−0.27 (0.19)	0.150
Income	−0.42 (0.25)	0.087	−0.12 (0.26)	0.643
Marital status	−0.93 (0.27)	≤0.001^*∗∗∗*^	−0.65 (0.29)	0.025^*∗*^

*Note. β* = the change in SCD while the variable increased by one unit, controlled for all other variables. SE = standard error. ^*∗*^=*p* < 0.05, ^*∗∗∗*^=*p* < 0.001.

**Table 4:** Effect of sleep quality, anxiety/depression, and worries on self-reported and other-reported SCD.

	Self-reported SCD		Other-reported SCD	
	*β* (SE)	*p* value	*β* (SE)	*p* value
Sleep quality	0.01 (0.02)	0.599	0.03 (0.03)	0.257
Anxiety/depression	0.31 (0.02)	≤0.001^*∗∗∗*^	0.30 (0.02)	≤0.001^*∗∗∗*^
Worries	0.21 (0.04)	≤0.001^*∗∗∗*^	0.24 (0.04)	≤0.001^*∗∗∗*^
Age	0.02 (0.02)	0.076	0.01 (0.01)	0.299
Gender	−0.15 (0.15)	0.316	−0.02 (0.16)	0.914
Education	0.08 (0.11)	0.457	−0.14 (0.12)	0.241
Income	−0.17 (0.15)	0.258	0.14 (0.17)	0.416
Marital status	−0.03 (0.17)	0.871	0.01 (0.19)	0.974

*Note. β* = the change in SCD while the variable increased by one unit, controlled for all other variables. SE = standard error. ^*∗∗∗*^=*p* < 0.001.

**Table 5:** Bootstrap result of serial mediation analysis for anxiety/depression and worries between sleep quality and SCD.

Paths	Self-reported SCD		Other reported SCD	
	*β* (SE)	*p* value	*β* (SE)	*p* value
Total effect	0.241 (0.037)	≤0.001	0.232 (0.039)	≤0.001
Direct effect	0.013 (0.024)	0.599	0.029 (0.026)	0.262
Total indirect effect	0.229 (0.035)^*∗*^	—	0.203 (0.039)^*∗*^	—
Serial mediation model 1				
Mediation effect (*X*^a^ ⟶ anxiety/depression ⟶ *Y*^b^)	0.155 (0.031)^*∗*^	—	0.128 (0.033)^*∗*^	—
Mediation effect (*X* ⟶ worries ⟶ *Y*)	0.045 (0.011)^*∗*^	—	0.046 (0.012)^*∗*^	—
Mediation effect (*X*^a^ ⟶ anxiety/depression ⟶ worries ⟶ *Y*^b^)	0.029 (0.008)^*∗*^	—	0.029 (0.009)^*∗*^	—
Serial mediation model 2				
Mediation effect (*X*^a^ ⟶ anxiety/depression ⟶ *Y*^b^)	−0.014 (0.022)	—	−0.012 (0.026)	—
Mediation effect (*X* ⟶ worries ⟶ *Y*)	0.074 (0.017)^*∗*^	—	0.075 (0.017)^*∗*^	—
Mediation effect (*X*^a^ ⟶ worries ⟶ anxiety/depression ⟶ *Y*^b^)	0.169 (0.024)^*∗*^	—	0.140 (0.028)^*∗*^	—

*Note.* ^a^ *X* = sleep quality; ^b^*Y* = SCD; ^*∗*^ = significant at the 0.05 level (two tailed).

## Data Availability

The data used to support the findings of this study are available from the corresponding author upon reasonable request.

## References

[1] Taylor C. A., Bouldin E. D., McGuire L. C. (2018). Subjective cognitive decline among adults Aged≥45 Years—United States, 2015–2016≥45 Years-United States, 2015-2016. *MMWR. Morbidity and mortality weekly report*.

[2] Wilmoth J., Bas D., Mukherjee S. (2023). World social report 2023: leaving no one behind in an ageing world. United Nations. https://desapublications.un.org/publications/world-social-report-2023-leaving-no-one-behind-ageing-world.

[3] He W., Goodkind D. (2016). *Paul Kowal U.S. Census Bureau, International Population Reports, P95/16-1, an Aging World: 2015*.

[4] Wang H., Chen H. (2022). Aging in China: challenges and opportunities. *China CDC weekly*.

[5] Jessen F., Amariglio R. E., van Boxtel M. (2014). A conceptual framework for research on subjective cognitive decline in preclinical Alzheimer’s disease. *Alzheimer’s and Dementia*.

[6] Buckley R. F., Maruff P., Ames D. (2016). Subjective memory decline predicts greater rates of clinical progression in preclinical Alzheimer’s disease. *Alzheimer’s and Dementia*.

[7] Mitchell A. J., Beaumont H., Ferguson D., Yadegarfar M., Stubbs B. (2014). Risk of dementia and mild cognitive impairment in older people with subjective memory complaints: meta-analysis. *Acta Psychiatrica Scandinavica*.

[8] Spira A. P., Chen-Edinboro L. P., Wu M. N., Yaffe K. (2014). Impact of sleep on the risk of cognitive decline and dementia. *Current Opinion in Psychiatry*.

[9] Kim J. H., Ahn J. H., Min C. Y., Yoo D. M., Choi H. G. (2021). Association between sleep quality and subjective cognitive decline: evidence from a community health survey. *Sleep Medicine*.

[10] Joo H. J., Joo J. H., Kwon J., Jang B. N., Park E. C. (2021). Association between quality and duration of sleep and subjective cognitive decline: a cross-sectional study in South Korea. *Scientific Reports*.

[11] Lee J. E., Ju Y. J., Park E. C., Lee S. Y. (2020). Effect of poor sleep quality on subjective cognitive decline (SCD) or SCD-related functional difficulties: results from 220,000 nationwide general populations without dementia. *Journal of Affective Disorders*.

[12] Zhao J. L., Cross N., Yao C. W. (2022). Insomnia disorder increases the risk of subjective memory decline in middle-aged and older adults: a longitudinal analysis of the Canadian Longitudinal Study on Aging. *Sleep*.

[13] Siddarth P., Thana-Udom K., Ojha R. (2021). Sleep quality, neurocognitive performance, and memory self-appraisal in middle-aged and older adults with memory complaints. *International Psychogeriatrics*.

[14] Exalto L. G., Hendriksen H. M. A., Barkhof F. (2022). Subjective cognitive decline and self-reported sleep problems: the SCIENCe project. *Alzheimer’s and Dementia*.

[15] Wang Z., Heizhati M., Wang L. (2022). Poor sleep quality is negatively associated with low cognitive performance in general population independent of self-reported sleep disordered breathing. *BMC Public Health*.

[16] Liew T. M. (2019). Depression, subjective cognitive decline, and the risk of neurocognitive disorders. *Alzheimer’s Research & Therapy*.

[17] Sánchez-Benavides G., Akinci M., Minguillón C. (2021). Subjective cognitive decline is associated with higher anxiety and depression during the COVID-19‒related confinement. *Alzheimer’s and Dementia*.

[18] Smith L., Shin J. I., Song T. J. (2022). Association between depression and subjective cognitive complaints in 47 low- and middle-income countries. *Journal of Psychiatric Research*.

[19] Gomzyakova N. A., Palchikova E. I., Tumova M. A., Kasyanov E. D., Sorokin M. Y. (2022). Association of anxiety and depression with objective and subjective cognitive decline in outpatient healthcare consumers with COVID-19: А cross-sectional study. *Consortium Psychiatricum*.

[20] Schwarz C., Lange C., Benson G. S. (2021). Severity of subjective cognitive complaints and worries in older adults are associated with cerebral amyloid-*β* load. *Frontiers in Aging Neuroscience*.

[21] Naismith S. L., Rogers N. L., Lewis S. J. (2011). Sleep disturbance relates to neuropsychological functioning in late-life depression. *Journal of Affective Disorders*.

[22] Mai E., Buysse D. J., Pandi-Perumal S. R., Monti J. M., Monjan A. A. (2009). Effect of depression and anxiety on sleep in the elderly. *Principles and Practice of Geriatric Sleep Medicine*.

[23] Hu Z., Zhu X., Kaminga A. C., Zhu T., Nie Y., Xu H. (2020). Association between poor sleep quality and depression symptoms among the elderly in nursing homes in Hunan province, China: a cross-sectional study. *BMJ Open*.

[24] Zhang J., Zhang Y., Luan Z., Zhang X., Jiang H., Wang A. (2020). A study on depression of the elderly with different sleep quality in pension institutions in Northeastern China. *BMC Geriatrics*.

[25] Liu X., Xia X., Hu F. (2022). The mediation role of sleep quality in the relationship between cognitive decline and depression. *BMC Geriatrics*.

[26] Wu C. R., Chen P. Y., Hsieh S. H. (2019). Sleep mediates the relationship between depression and cognitive impairment in older men. *American Journal of Men’s Health*.

[27] Babson K. A., Trainor C. D., Feldner M. T., Blumenthal H. (2010). A test of the effects of acute sleep deprivation on general and specific self-reported anxiety and depressive symptoms: an experimental extension. *Journal of Behavior Therapy and Experimental Psychiatry*.

[28] Xu W. Q., Lin L. H., Ding K. R. (2021). The role of depression and anxiety in the relationship between poor sleep quality and subjective cognitive decline in Chinese elderly: exploring parallel, serial, and moderated mediation. *Journal of Affective Disorders*.

[29] Xiao S., Shi L., Zhang J. (2023). The role of anxiety and depressive symptoms in mediating the relationship between subjective sleep quality and cognitive function among older adults in China. *Journal of Affective Disorders*.

[30] Liu J., Li S., Yan X. (2023). Social connection and lifestyle factors associated with happiness in urban older adults in China: a cross-sectional study with a community sample. *Research in Gerontological Nursing*.

[31] Achenbach T. M., Newhouse P. A., Rescorla L. A. (2004). *Manual for the ASEBA Older Adult Forms & Profiles*.

[32] Ivanova M. Y., Achenbach T. M., Rescorla L. A. (2020). The generalizability of Older Adult Self-Report (OASR) syndromes of psychopathology across 20 societies. *International Journal of Geriatric Psychiatry*.

[33] Zheng B., Li M., Wang K. L., Lv J. (2016). Analysis of the reliability and validity of the Chinese version of Pittsburgh sleep quality index among medical college students. *Beijing da xue xue bao. Yi xue ban = Journal of Peking University. Health sciences*.

[34] Zhang C., Zhang H., Zhao M. (2020). Reliability, validity, and factor structure of Pittsburgh sleep quality index in community-based centenarians. *Frontiers in Psychiatry*.

[35] Yan D. Q., Huang Y. X., Chen X., Wang M., Li J., Luo D. (2021). Application of the Chinese version of the Pittsburgh sleep quality index in people living with HIV: preliminary reliability and validity. *Frontiers in Psychiatry*.

[36] Zhang Q., Sun M. A., Sun Q., Mei H., Rao H., Liu J. (2023). Mental fatigue is associated with subjective cognitive decline among older adults. *Brain Sciences*.

[37] Coutts J. J., Hayes A. F. (2022). Questions of value, questions of magnitude: an exploration and application of methods for comparing indirect effects in multiple mediator models. *Behavior Research Methods*.

[38] Zhu X., Hu Z., Nie Y. (2020). The prevalence of poor sleep quality and associated risk factors among Chinese elderly adults in nursing homes: a cross-sectional study. *PLoS One*.

[39] Park J. H., Yoo M. S., Bae S. H. (2013). Prevalence and predictors of poor sleep quality in Korean older adults. *International Journal of Nursing Practice*.

[40] Yu J., Rawtaer I., Fam J. (2016). Sleep correlates of depression and anxiety in an elderly Asian population. *Psychogeriatrics*.

[41] Paudel M. L., Taylor B. C., Diem S. J. (2008). Association between depressive symptoms and sleep disturbances in community-dwelling older men. *Journal of the American Geriatrics Society*.

[42] Hill N. L., Bhargava S., Bratlee-Whitaker E., Turner J. R., Brown M. J., Mogle J. (2021). Longitudinal relationships between subjective cognitive decline and objective memory: depressive symptoms mediate between-person associations. *Journal of Alzheimer’s Disease*.

[43] Lu S., Reavley N., Zhou J. (2018). Depression among the general adult population in Jiangsu Province of China: prevalence, associated factors and impacts. *Social Psychiatry and Psychiatric Epidemiology*.

[44] Schlosser M., Demnitz-King H., Whitfield T., Wirth M., Marchant N. L. (2020). Repetitive negative thinking is associated with subjective cognitive decline in older adults: a cross-sectional study. *BMC Psychiatry*.

[45] Shokri-Kojori E., Wang G.-J., Wiers C. E. (2018). *β*-Amyloid accumulation in the human brain after one night of sleep deprivation. *Proceedings of the National Academy of Sciences*.

